# Impact of Cold Waves on Cognitive Functions in Older Adults: A Mixed-Methods Study in Eastern China

**DOI:** 10.1093/geroni/igaf122.2706

**Published:** 2025-12-31

**Authors:** Qingwei Wang, Yuewen Wu, Yue Zhou

**Affiliations:** Xi’an Jiaotong-Liverpool University, Suzhou, Jiangsu, China (People’s Republic); Xi’an Jiaotong-Liverpool University, Suzhou, Jiangsu, China (People’s Republic); Xi’an Jiaotong-Liverpool University, Suzhou, Jiangsu, China (People’s Republic)

## Abstract

**Background:**

As global temperatures become more unpredictable, extreme weather like heat waves and cold waves are increasingly common, creating serious health risks for older adults. Given many studies have focused on the effects of heat waves on mortality, the impact of cold waves on cognitive well-being remains unexplored.

**Methodology:**

This study employs a mixed-methods approach to examine the impact of cold waves on cognitive performance in older adults. The quantitative analysis explores correlations using data from 3,970 individuals aged 60+ in Jiangsu, Zhejiang, and Shanghai from the China Health and Retirement Longitudinal Study (CHARLS). To further identify and examine causative factors, the qualitative component includes semi-structured interviews with 11 older adults at Shanghai Yangjing Daycare Center, supplemented by observations and video recordings. By first establishing statistical correlations and then exploring causative factors through qualitative insights, this study offers a methodologically innovative and comprehensive understanding of cold waves’ effects on cognitive well-being.

**Findings:**

Two studies highlighted the key roles of medical insurance, social engagement, and exercise in reducing cognitive decline among older adults during cold waves. The qualitative component further identified indoor exercise as an effective coping strategy and emphasizing the need for widely covered heating infrastructure, offering valuable insights for policy interventions despite some inconsistencies in individual responses.

**Conclusion:**

This study reveals the negative impact of cold waves on older adults’ cognitive function, urging interventions to properly overcome the shortage of heating, reduced social interactions and outdoor exercises during cold waves, and finally, building their body resilience to non-optimal temperatures.

